# *bantam* Is Required for Optic Lobe Development and Glial Cell Proliferation

**DOI:** 10.1371/journal.pone.0032910

**Published:** 2012-03-08

**Authors:** Ying Li, Richard W. Padgett

**Affiliations:** Department of Molecular Biology and Biochemistry, Waksman Institute, Cancer Institute of New Jersey, Rutgers University, Piscataway, New Jersey, United States of America; VIB & Katholieke Universiteit Leuven, Belgium

## Abstract

microRNAs (miRNAs) are small, conserved, non-coding RNAs that contribute to the control of many different cellular processes, including cell fate specification and growth control. Drosophila *bantam*, a conserved miRNA, is involved in several functions, such as stimulating proliferation and inhibiting apoptosis in the wing disc. Here, we reported the detailed expression pattern of *bantam* in the developing optic lobe, and demonstrated a new, essential role in promoting proliferation of mitotic cells in the optic lobe, including stem cells and differentiated glial cells. Changes in *bantam* levels autonomously affected glial cell number and distribution, and non-autonomously affected photoreceptor neuron axon projection patterns. Furthermore, we showed that *bantam* promotes the proliferation of mitotically active glial cells and affects their distribution, largely through down regulation of the T-box transcription factor, *optomotor-blind (omb, Flybase, bifid)*. Expression of *omb* can rescue the *bantam* phenotype, and restore the normal glial cell number and proper glial cell positioning in most Drosophila brains. These results suggest that *bantam* is critical for maintaining the stem cell pools in the outer proliferation center and glial precursor cell regions of the optic lobe, and that its expression in glial cells is crucial for their proliferation and distribution.

## Introduction

microRNAs (miRNAs) are an evolutionarily conserved, abundant class of small, non-coding RNAs, which are about 22 nucleotides in length. To date, 1424 miRNAs have been identified in the human genome and 238 miRNAs in *Drosophila melanogaster* (www.mirbase.org), although the function for most of them has not been elucidated. Each miRNA is thought to target multiple genes in the genomes, and many genes are thought to be partially regulated by one or more miRNAs. In humans, over one-third of our genes are predicted to be directly targeted by miRNAs [1]. In metazoans, miRNAs typically down regulate gene expression by binding to complementary sequences in the 3′ untranslated regions (3′ UTR) of their target mRNAs, usually resulting in inhibition of protein translation. miRNAs are known to play widespread and critical roles in a variety of cellular processes including proliferation, differentiation, apoptosis, development, and tumor progression [2], [3], [4]. Numerous miRNAs have been reported to be expressed in a spatially and temporally controlled manner in the nervous system, suggesting their important roles in brain function and development [5], [6], [7], [8].

*bantam* is a conserved miRNA originally discovered in Drosophila that is expressed in a spatio-temporally restricted manner throughout development [9], [10]. It was originally identified in a gain-of-function screen for genes that stimulate tissue growth [10]. Further work has showed that *bantam* plays important roles in many different processes and functions during development. By targeting the pro-apoptotic gene *head involution defective* [9], *bantam* plays a role in modulating ionizing radiation-induced apoptosis [11]. In the adult ovary, *bantam* is required for germline stem cell (GSC) maintenance [12], [13]. In the Drosophila nervous system, *bantam* inhibits polyQ- and tau-induced neurodegeneration [14], [15]. In the central nervous system (CNS), *bantam* targets *clock*, a core circadian clock gene that regulates circadian rhythms [16]. In the peripheral nervous system (PNS), *bantam* functions in epithelial cells to non-autonomously regulate scaling growth of class IV dendrites of dendrite arbor (da) sensory neurons [17]. Given that miRNAs are abundantly expressed in the brain, including *bantam*, the question arises what role *bantam* plays in the function of the Drosophila brain.

We examined the possible role of *bantam* in the Drosophila visual system, which is composed of a pair of compound eyes and the optic ganglia. The compound eyes are composed of ∼800 repeated units, called ommatidia. Each of these units contains eight photoreceptor neurons (R1–R8 neurons) and a complement of non-neural support cells arranged in an invariant pattern. During larval development, axons from photoreceptors in the eye disc project through the optic stalk into different layers of the optic lobe. The optic lobes are the visual processing centers of the brain and include three ganglia—the lamina, medulla, and lobula complexes. Axons from photoreceptor R1–R6 neurons project between two layers of lamina glial cells, the epithelial and marginal glia, and form the lamina plexus while R7 and R8 neurons connect to a deeper target site known as the medulla [18], [19]. The outer proliferation center (OPC) and inner proliferation center (IPC) are contained in the Drosophila optic lobe. In the OPC, a small group of mitotically active progenitor cells, which are located anterior to the lamina furrow on the surface of the optic lobe, give rise to the lamina precursor cells (LPCs). Once they are posterior to the lamina furrow, LPCs divide to produce lamina neurons. The OPC progenitor cells close to the central brain are responsible for producing outer medulla neurons while IPC cells generate inner medulla and lobula neurons.

Glial cells and neurons have an intimate association in the brain, but have distinct origins. In Drosophila, glial cells are normally classified by their relative position and morphology [20]. In the third instar larval optic lobe, the epithelial, marginal, and medulla glial cells are organized into three rows around the border of the lamina and medulla. In the medulla, medulla neuropil glial cells enwrap the axons and separate the medulla cortex from the central brain. Lamina epithelial and marginal glial cells are generated from glial precursor cell (GPC) areas at the tips of the OPC, located at the prospective dorsal and ventral margins of the optical lobe [20]. However, which genes regulate the differentiation, proliferation, and migration of glial cells is not well understood.

Fly and vertebrate visual systems share similar features of organization, including the stereotyped retinotopic map. With accessibility to genetic, molecular, and behavior tools, Drosophila has been a powerful model system for studying the underlying mechanisms controlling axonal pathfinding and glial cell development. Studies from the fly often shed light on its more complicated vertebrate counterparts.

In this paper, we report the detailed expression pattern of *bantam* in the optic lobe of the third instar larval brain, and show that it is required for maintaining stem cell pools in the OPC and GPC regions of the optic lobe. Glial cell expression of *bantam* is crucial for glial cell proliferation and distribution. Our results also showed that *bantam* autonomously affects glial cell number and distribution, and non-autonomously affects photoreceptor axon projection patterns. The function of *bantam* on glial cells is largely dependent on its down regulation of the T-box transcription factor, *omb*.

## Results

### *bantam* is highly expressed in mitotically active cells in the optic lobe

*bantam* has been previously shown to function in cell proliferation in the wing and eye disc [9], [21], which suggests it may play a broader role in proliferation. The optic lobe in Drosophila undergoes rapid cell division and requires stepwise control to ensure a precisely coordinated assembly of the visual system. To study the function of *bantam* in the development of the visual system, we first examined *bantam* expression patterns in the optic lobe of third instar larval brains. To gauge the expression of miRNAs, sensor constructs are commonly used. These are designed with two copies of a perfect target sequence, in this case *bantam* binding sites, introduced in the 3′UTR of a green fluorescent protein (GFP) construct. If *bantam* is expressed in cells containing this sensor, then the perfect binding of the miRNA to the 3′UTR will result in degradation of the GFP mRNA and consequently the intensity of GFP. This construct is therefore used as the negative indicator of *bantam* expression levels [9].

**Figure 1:**
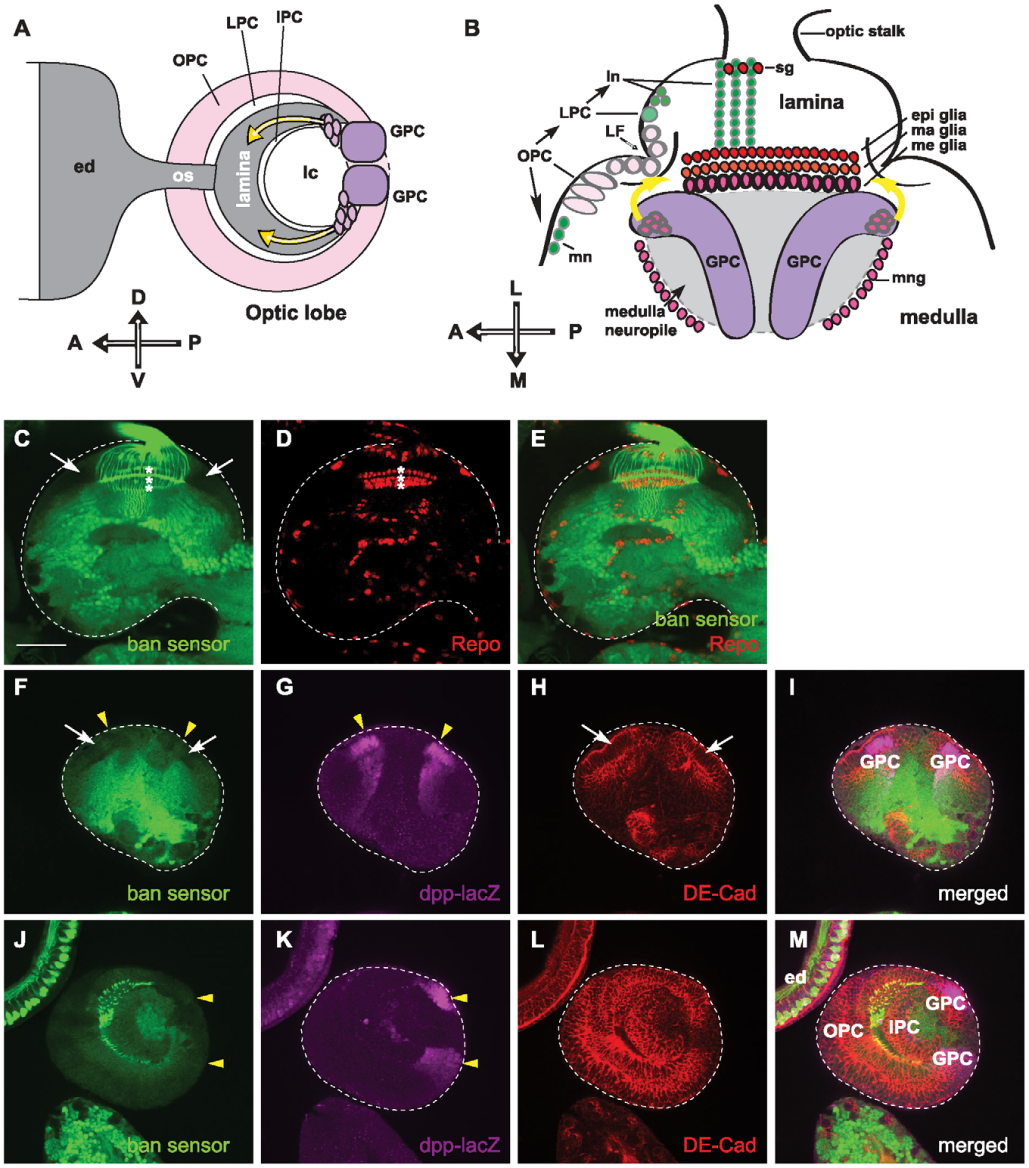
*bantam* is differentially expressed within the optic lobe. (**A, B**) The schematic diagrams illustrate the third instar larval visual system. (**A**) Lateral view with anterior left, posterior right, dorsal up, and ventral down. All brains are oriented in the same direction in the lateral and horizontal views in all the figures. Photoreceptor neuron axons from the eye disc (ed) project through the optic stalk (os) into the optic lobe (crescent shape in gray). Glial precursor cell (GPC) regions are labeled in magenta. Yellow arrows indicate the migrating paths of lamina glial cells from GPC to the lamina target region. The outer proliferation center (OPC); the lamina precursor cell (LPC); the inner proliferation center (IPC); and the lobula complex (lc) are labeled. (**B**) Horizontal views show anterior left, posterior right, and lateral up. Neuroblasts in the OPC closest to the lamina furrow (LF) give rise to the LPC, which in turn divides to produce lamina neurons (ln). Neuroblasts in the OPC close to the medulla generate medulla neurons (mn). Three layers of lamina glial cells set the boundary of the lamina and medulla. Subtypes of glia are labeled and include: satellite glia (sg), epithelial glia (epi glia), marginal glia (ma glia), medulla glia (me glia), and medulla neuropil glia (mng). GPC areas are located at the prospective dorsal and ventral margins of the optical lobe at the superficial focal plane of horizontal view. Yellow arrows indicate the migrating paths of lamina glial cells from GPC to the lamina target region under the lamina furrow. (**C–E**) Shown is one focal plane of a horizontal view, where three rows of lamina glial cells are present. The dashed line outlines the brain. (**C**) The *bantam* sensor (green) shows high expression levels in photoreceptor axons and neurons in the medulla, but very low expression levels in OPC cells (white arrows) and lamina glial cells (asterisks). (**D**) Glial cells are labeled by anti-Repo staining (red). Three rows of laminal, epithelial, marginal and medulla glial cells are visible (asterisks). (**E**) merged images. (**F–I**) Shown is one focal plane of a superficial horizontal view, where the GPC regions are present. (**F**) The *bantam* sensor (green) shows low expression levels in the OPC (white arrows) and GPC regions (solid yellow arrowheads). (**G**) The GPC regions are labeled by *dpp-lacZ* and stained for β-galactosidase (magenta)(noted by solid yellow arrowheads). (**H**) Anti-DE-cadherin is labeled (red) to view the neuroepithelial cells in the OPC (white arrows). (**I**) merged images. (**J–M**) Shown is one focal plane of a lateral view, where the GPC, OPC and IPC are visible. (**J**) The *bantam* sensor (green) shows high expression levels in the photoreceptor neurons of the eye discs, and low expression levels in the OPC, IPC and GPC regions. (**K**) GPC regions (solid yellow arrowheads) are located at the dorsal and ventral margin of the posterior optic lobe, and were labeled by *dpp-lacZ* and stained for β-galactosidase (magenta). (**L**) Anti-DE-cadherin is labeled (red) to view the neuroepithelial cells in the optic lobe. (**M**) merged images. Scale bar: 50 µm.

To distinguish the structure of the optic lobe, we used antibody staining to view different subtypes of cells. DE-cadherin (DE-cad) is a transmembrane protein located at the zonula adherens between epithelial cells and marks the cell surface. We used anti-DE-cadherin to view optic lobe neuroepithelia (Figure 1H, 1L), which act as progenitors of optic lobe neuroblasts in the OPC and the IPC [22]. *decapentaplegic (dpp)* is expressed in the GPC regions of the optic lobe [23], [24], and a Dpp-lacZ enhancer trap line [25] was used to visualize the GPC region marker (Figure 1G, 1K). Reversed polarity (Repo) is a glial specific homeodomain protein expressed in all glial-cell subtypes in the visual system [26], [27]. We used anti-Repo staining to view differentiated glial cells in the optic lobe (Figure 1D). By examining *bantam* sensor expression in the third instar larval visual system, we found that *bantam* is expressed differentially in the third larval optic lobe. *bantam* sensors displayed low expression levels in the neuroepithelial cells of the OPC (white arrows in Figure 1C,1F, 1I), cells at the GPC areas (yellow solid arrows in Figure 1F, 1I, 1J, 1M), and also in the mature glial cells (asterisks in Figure 1C, 1E), which indicates high *bantam* expression levels in those cells (Figure 1). The *bantam* sensor showed high expression in differentiated neurons in the optic lobe, and in the photoreceptor neuron cells in the eye discs (Figure 1C, 1J). This indicates that *bantam* expression levels are low in those cells. Previous studies also found low *bantam* levels in the photoreceptor neurons [28].

### *bantam* is required for proliferation in the optic lobe

**Figure 2:**
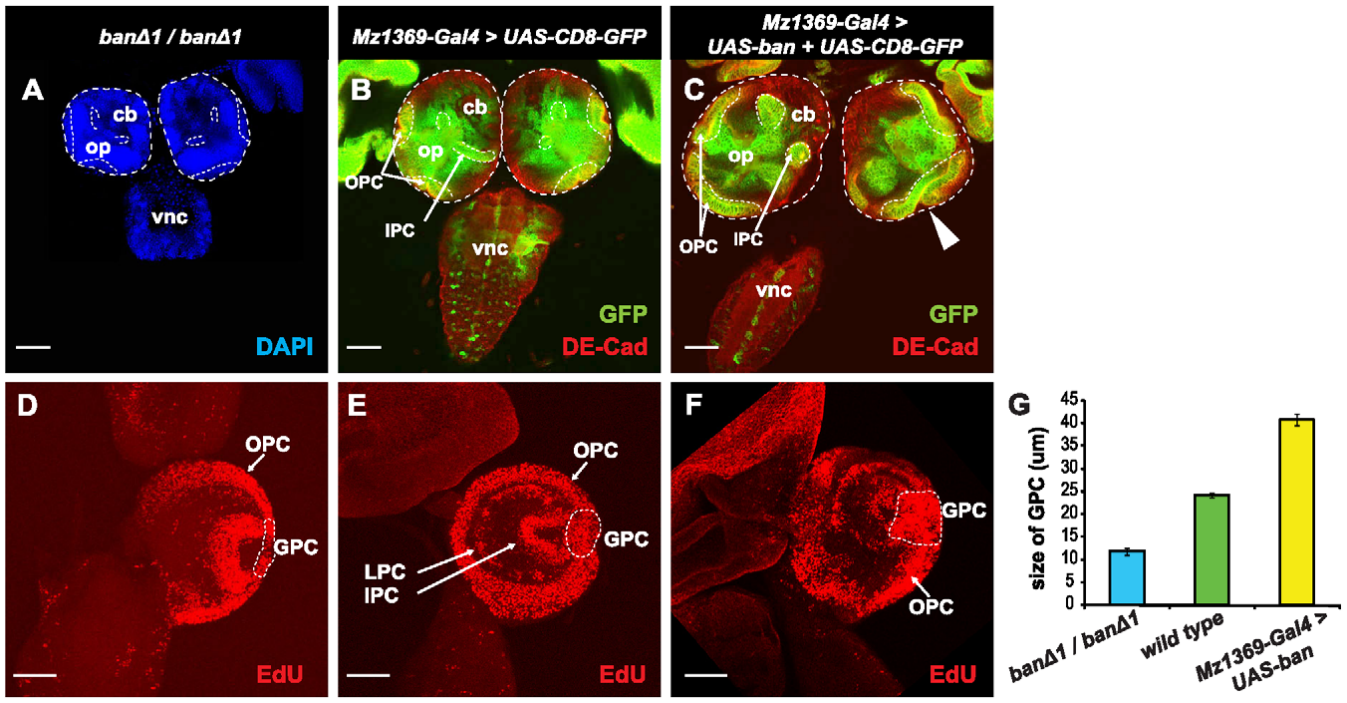
*bantam* is required for proliferation in the optic lobe. (**A, B, C**) Brains are positioned with a horizontal view on a similar, single focal plane, and images were taken at the same magnification. A dashed line for brain size comparison outlines the brain surface. (**A**) A *ban^Δ1^/ban^Δ1^* null mutant is shown. The brain is stained with DAPI (blue) in order to view all the cells. (**B, C**) *UAS*-CD8-GFP (green) is used to view the expression of Mz1369-Gal4 in the optic lobe. DE-cadherin staining (red) is used to view neuroepithelial cells in the OPC and IPC. Part of the OPC and IPC can been seen at this focal plane, and outlined by the dashed line. op, optic lobe; cb, central brain; vnc, ventral nerve cord. (**B**) wild type brains; (**C**) Over expression of *bantam* causes a broader size of the optic lobe and folded neuroepithelia (white arrowhead). (**D–F**) The brains are positioned for a lateral view. The projection images are from multiple section planes that cover all proliferation centers in the optic lobe. EdU staining (red) shows cell proliferation in the brain. DAPI (blue) is used to view the outline of the brain. The OPC, LPC, and IPC are labeled with white arrows, and the GPC is outlined with a white dashed line. Genotypes: (**D**) *ban^Δ1^/ban^Δ1^*; (**E**) *UAS*-CD8-GFP/+; Mz1369-Gal4/+; (**F**) *UAS*-CD8-GFP/+; Mz1369-Gal4/*UAS-ban*. (G) histograms of the diameter of the GPC region in the optic lobe of third-instar larvae. The measurements were taken in the circled areas in D, E, and F; *ban^Δ1^/ban^Δ1^* (11.82±0.78 µm, n = 6), wild type (24.27±0.45 µm, n = 12), Mz1369-Gal4>*UAS-ban* (40.93±1.2 µm, n = 14). Scale bar: 50 µm.

*bantam* has been reported to promote growth in the wing and eye tissues [9], [21]. We found that *bantam* is highly expressed in the OPC, GPC areas, and glial cells in the optic lobe, where cells are mitotically active. This led us reason that *bantam* might be critical in those cells for cell proliferation in the developing brain. To test this hypothesis, we first examined the brain size of the wild type, *bantam* null mutant, and over-expressed *bantam*. *ban^Δ1^* is a null allele resulting from a *bantam* gene deletion [10]. Homozygous *ban^Δ1^/ban^Δ1^* brains (Figure 2A) showed a smaller size compared to wild-type brains (Figure 2B). We used an optic lobe driver, Mz1369-Gal4 [29], to express *UAS*-CD8-GFP, a fusion-GFP protein expressed at the cell membrane, in order to distinguish the optic lobe from the central brain. When *bantam* was over expressed in the optic lobe by the Mz1369-Gal4 driver (Figure 2C), a bigger brain was observed compared to wild-type brains. This was due to both the expansion of the optic lobe and to the expansion of folded neuroepithelial cells (Figure 2C). Neuroepithelial cells are the main component of the OPC region in the developing optic lobe [22]. To determine how cell proliferation is affected, EdU staining was performed, which is an alternative to traditional BrdU staining for detecting newly synthesized DNA. Wild-type animals have stereotype proliferation patterns at the third instar larval stage, showing active proliferation in the OPC, LPCs, the IPC, and GPC regions (Figure 2E). We did not see much change in the proliferation patterns in the optic lobes of *bantam* null mutants or in animals over expressing *bantam*, but did notice that the size of the GPC region was greatly affected by changes of *bantam* levels (Figure 2G). Losing *bantam* in homozygous *ban^Δ1^/ban^Δ1^* animals (Figure 2D) led to a significantly thinner GPC region (Figure 2G, p-value<0.0001), compared to the wild type (Figure 2E). When *bantam* was over expressed in the optic lobe (Figure 2F), a dramatic increase of EdU staining in the GPC region was increased (Figure 2G, p-value<0.0001), compared to the wild type. All these results indicate that *bantam* is required for cell proliferation in the OPC and GPC regions of the optic lobe.

### *bantam* acts in the brain for proper R axon projection patterns

**Figure 3:**
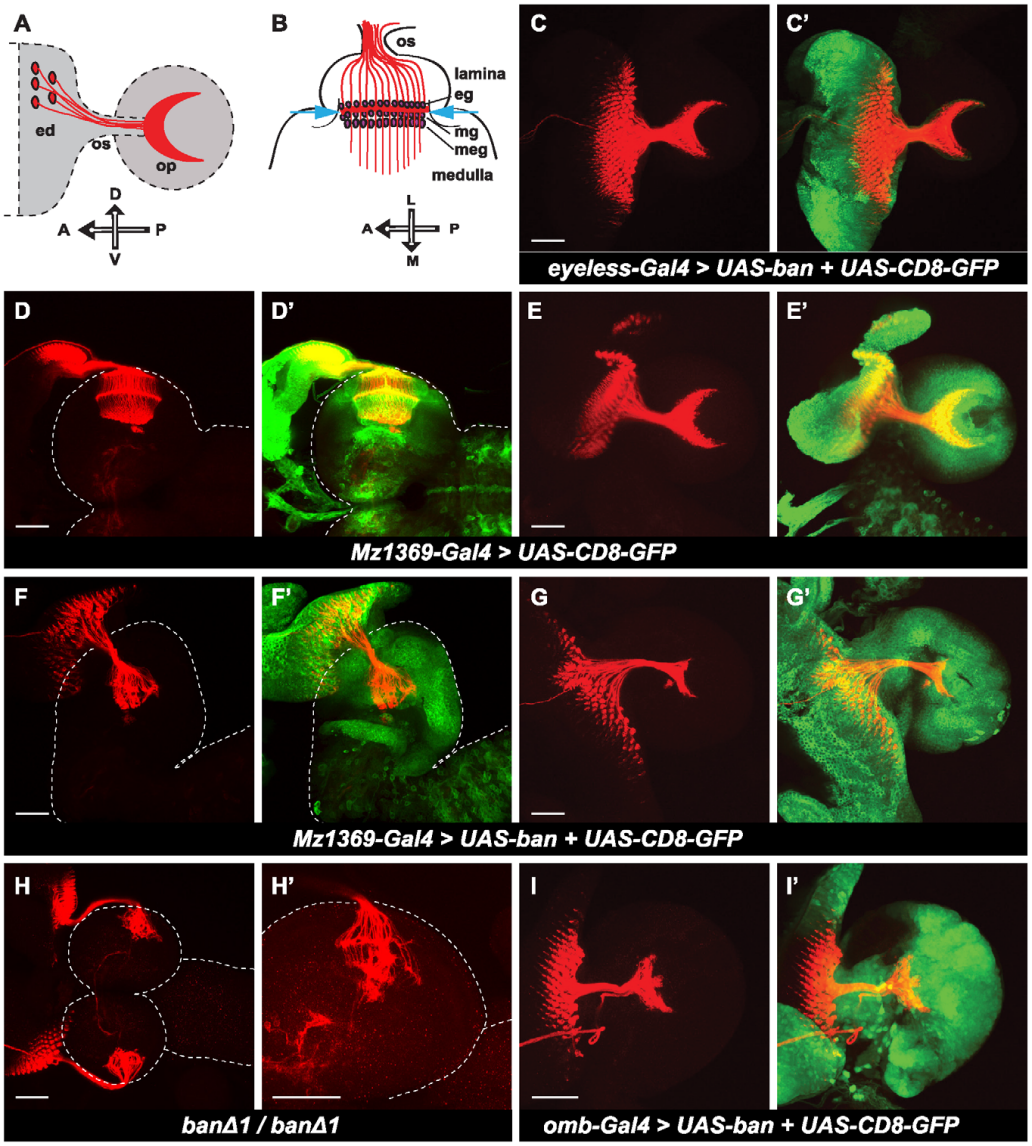
*bantam* affects photoreceptor-neuron axon projection in the optic lobe. (**A, B**) Schematic illustration of the photoreceptor (R1–R8) axon projection patterns in the late third instar larval brain of Drosophila. (**A**) A lateral view of axons from photoreceptor neurons (R1–R8) (red) in the eye disc (ed) projecting through the optic stalk (os) into the optic lobe (op). The projection pattern of R axons in the optic lobe is crescent shaped (red). (**B**) A horizontal view of axons (red) of R cells projecting into different layers of the optic lobe. Axons from R1–R6 (red) stop between two layers of glial cells in the lamina, the epithelial (eg) and marginal glial cells (mg) (magenta), and form the lamina plexus (red line between two blue arrows). R7 and R8 project deeper into the medulla (A: anterior; P: posterior; D: dorsal; V: ventral; L: lateral; M: middle). The anti-Chaoptin (red) was used to view R-cell projection patterns. *UAS*-CD8-GFP (green) was used to visualize expression patterns of Gal4 drivers. Brain surface is outlined by dashed lines. (**D, D′, F, F′, H, H′**) Brains are positioned for a horizontal view. (**C, C′, E, E′, G, G′, I, I′**) Brains are positioned for a lateral view. (**C, C′**) over expression of *bantam* by the eye-specific driver, *eyeless-Gal4*. (**D–E′**) wild type brains. (**F–G′**) over expression of *bantam* by the optic lobe driver, Mz1369-Gal4. (**H, H′**) *ban^Δ1^/ban^Δ1^* null mutants. (**H**) Lower magnification shows the two brain hemispheres. (**H′**) Higher magnification only showing half of a hemisphere. (**I, I′**) Over expression of *bantam* by *omb-Gal4*. Scale bar: 50 µm.

The OPC and GPC regions are sources of progenitor cells for neurons and glial cells for the optic lobe. Because *bantam* is highly expressed in these regions, and is also important for proliferation, we reasoned that expression level changes of the genes affecting proliferation in these regions might affect the pool of neural stem cells. It might eventually affect the final number of differentiated neurons and glia, causing an abnormal structure of the optic lobe, and therefore affect the photoreceptor neuron (R neuron) axon projection pattern in the optic lobe. We first examined the R axon projection patterns in the optic lobe by modulating *bantam* expression. We used anti-Chaoptin to visualize R axons. In the horizontal view of the wild-type third instar larval brain (Figure 3D, 3D′), R axon fibers are finely spaced by the lamina neurons, and R1–R6 are terminated at the bottom of lamina between two rows of glial cells, epithelial and marginal glial cells, and their growth cones form the linear lamina plexus. R7 and R8 axons project deeper into medulla, forming a lattice-like network. In *ban^Δ1^/ban^Δ1^* larval brains (Figure 3H, 3H′), R projection patterns were disrupted, varying from intermediate to severe degrees of disruption. In the severe cases, R axons appeared in thick bundles, and stopped in the brain irregularly. In the intermediate cases, there were visible lamina plexuses, which did not appear evenly linear, and became shorter with occasional breaks. Projections in the medulla were also disrupted. In addition, when *bantam* was over expressed by Mz1369-Gal4 (Figure 3F, 3F′), we found similarly disrupted R axon projection patterns like those observed in *ban^Δ1^/ban^Δ1^* larval brains, even though Mz1369-Gal4>*ban* brains showed a bigger size (compare Figure 3F, 3H). These results indicate that *bantam* is required for maintaining the correct R axon projection patterns.

Altered R axon projection patterns might be the result of the change of genes affecting axon pathfinding, or a secondary effect of the disruption of the integrity of the brain structure. To determine where *bantam* acts to cause this phenotype, we compared the R axon projection patterns when different Gal4 drivers were used to over express *bantam*. Mz1369-Gal4 and *omb*-Gal4 are both expressed not only in the optic lobe, but also in the eye discs, while *eyeless*-Gal4 is only expressed in the eye discs, and not in the optic lobe. In the lateral view of the wild-type larval brain (Figure 3E, 3E′), R axon projection patterns appear in a crescent-like shape. When *bantam* was over expressed in the optic lobe by the Mz1369-Gal4 driver, the crescent shape of the R axon projection pattern was disrupted (Figure 3G, 3G′). Similarly, disrupted R axon projection patterns were also seen when *bantam* was over expressed with a different optic-lobe driver, *omb*-Gal4 (Figure 3I, 3I′). But when *bantam* was over expressed by *eyeless*-Gal4 (Figure 3C, 3C′), we observed that R axon projection patterns appeared like wild type, although an overgrowth of eye discs was present. This indicates that *bantam* is acting in the optic lobe but not in R neurons for the correct R axon projection.

### *bantam* acts in glial cells but not in neurons

**Figure 4:**
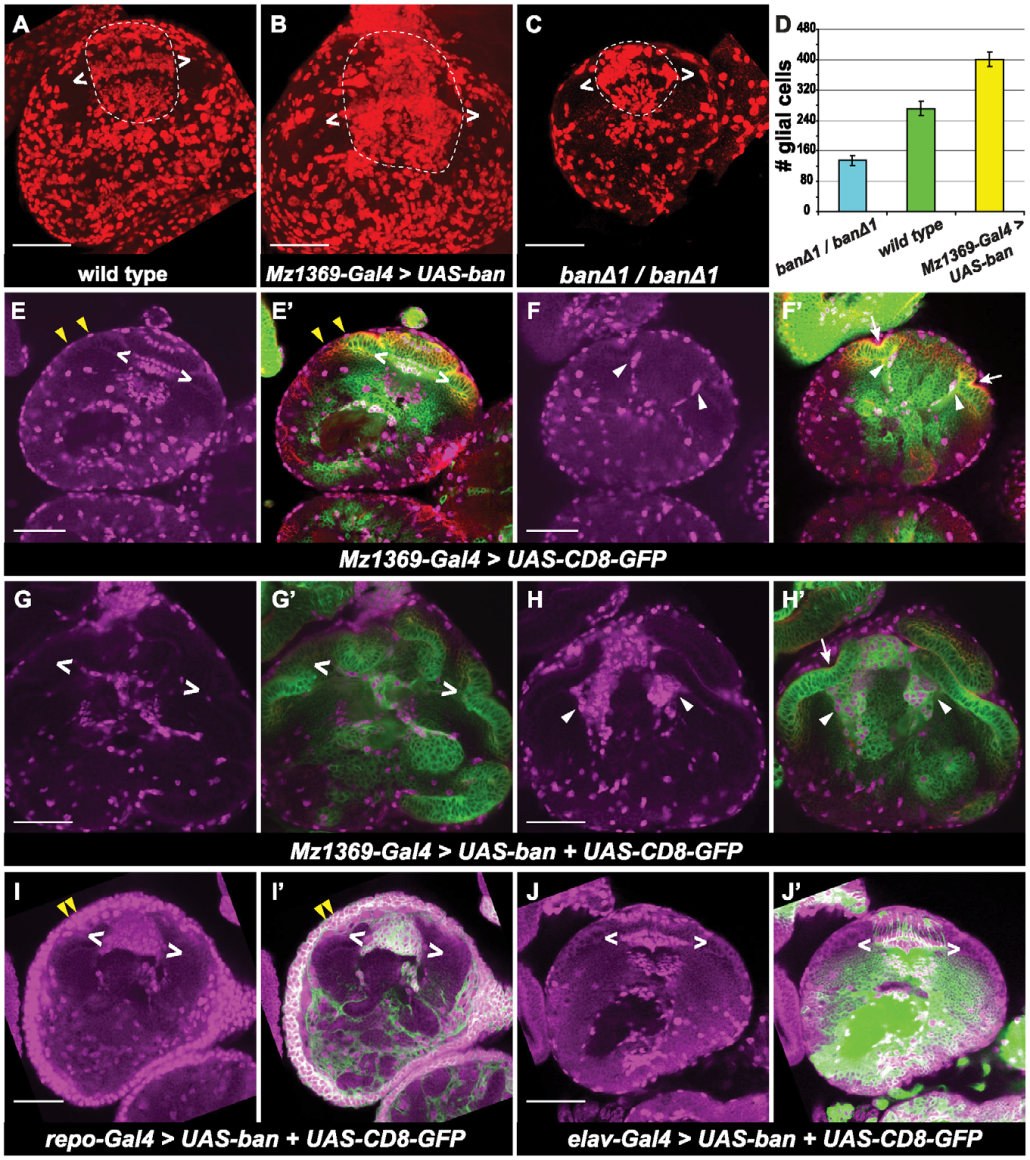
*bantam* promotes glial cell proliferation in the optic lobe. All brains are positioned for horizontal views. (**A–C**) Maximum projection to view total number of glial cells which are stained for anti-Repo (red). Glial cells in the lamina and medulla are circled inside the dashed line. Glial cells between brackets (two white < >) correspond to the three layers of laminal glial cells: epithelial, marginal and medulla glia. (**A**) wild type brains. (**B**) over expression of *bantam* by Mz1369-Gal4. (**C**) *ban^Δ1^/ban^Δ1^* null mutant. (D) histograms of glia number in the optic lobe of the third instar larvae. Glia number were counted in the region circled in A, B, and C. *ban^Δ1^/ban^Δ1^* (135±13, n = 5); wild type (272±18, n = 4); Mz1369-Gal4>UAS-ban (402±20, n = 4). (**E–J′**) A single focal plane is shown. Glial cells are stained by anti-Repo (magenta). *UAS*-CD8-GFP (green) is used to view the expression of Gal4. Neuroepithelial cells are viewed by anti- DE-cadherin (red). (**E–F′**) wild type. (**G–H′**) over expression of *bantam* by the optic lobe driver, Mz1369-Gal4. (**I, I′**) over expression of *bantam* by the glia driver, *repo-Gal4*. (**J, J′**) over expression of *bantam* by the neuron driver, *elav-Gal4*. (**E, E′**), (**G, G′**), (**I, I′**) and (**J, J′**) are at a similar focal plane, where three rows of lamina glial cells are present. (**F, F′**) and (**H, H′**) are at a similar focal plane, where the lamina furrow (white arrows) and migrating glia (white arrowheads) are present. Cell surface glia cells are indicated by yellow arrow heads. Scale bar: 50 µm.

In the developing optic lobe, R axons and glial cells affect each other in order to maintain the integrity of the visual system. Migration of lamina glial cells depends on the local signaling from R axons [30]. Lamina glial cells function as intermediate targets of R1–R6 axons and are required for establishing the correct R axon projection pattern [19], [31]. In *bantam* null mutants and in animals over-expressing *bantam* in the visual system, we did not see much change in the number of R cells (Figure 3H, 3G), but an altered R axon projection was observed. We reasoned that the altered R axon projection patterns in those cases might be the cause of a change in glial cell number and/or distribution in the optic lobe. Therefore, we examined the number and distribution of glial cells in the optic lobe under these experimental conditions. In wild-type 3rd instar larval optic lobes, there are four main subtypes of glial cells based on their positions: 1) surface glia on the surface of the brain, 2) satellite glia in the lamina, 3) three distinct layers of glia called epithelial, marginal, and medulla glial cells at the lamina-medulla boundary, and 4) medulla neuropil glia in the medulla (Figure 4A, 4E). To exam whether *bantam* affects glial cell numbers in the optic lobe, we quantified the total glial cells present, except for the surface glia in the optic lobe. In *bantam* null mutant larval brains (Figure 4C), total glial cell number was significantly reduced (Figure 4D, p-value<0.0002) compared to the wild type (Figure 4A). When *bantam* was over expressed (Figure 4B), the total glial cell number significantly increased (Figure 4D, p-value<0.0016) compared to the wild type. We also checked the effect of *bantam* on glial cell distribution. In wild type, at the boundary of the lamina and medulla glial cells were well organized into three layers (Figure 4E). When *bantam* was over expressed, the distribution of mature glial cells was disturbed, in that the three lamina glia layers were not clearly distinguishable, and less glial cells were present around the lamina plexus (Figure 4G). In the wild type, lamina epithelial and marginal glial cells were produced in GPC regions, and migrated under the lamina furrow to their final destination [30]. Therefore, only a few glial cells were present under the lamina furrow at a single focal plane in wild type (Figure 4F). However, when *bantam* was over expressed by Mz1369-Gal4, there were many glial cells present under the lamina furrow (Figure 4H). This effect was not specific to Mz1369-Gal4 because a different Gal4 line, *omb*-Gal4, gave a similar result (Figure S1). All of these findings indicate that *bantam* is important for regulation of glial cell number and organization in the optic lobe.

Because Mz1369-Gal4 and *omb*-Gal4 are expressed both in neurons and glial cells, we cannot tell which type of cells *bantam* is acting in to cause the change in glial cell numbers and distribution. To determine this, we used cell-type specific Gal4 lines, which are expressed only in glial cells or neurons, and then checked glial cell number and organization. *repo*-Gal4 is expressed in all differentiated glial cells, but not in neurons. When *bantam* was over expressed by *repo*-Gal4 (Figure 4I, 4I′), brains were slightly larger because of a dramatic increase in the number of glial cells. At the surface of the brain, increased glial cells made multiple layers, forming a thicker glial sheath. On the border of the lamina and medulla, the three layers of glial cells were not able to be distinguishable. Large ectopic glial cell clusters were seen in the lamina. The *elav*-Gal4 driver strongly expresses in neurons, but not in glial cells. When *bantam* was over expressed by *elav*-Gal4 (Figure 4J, 4J′), the brain remained wild-type size, and there was a normal glial cell distribution observed along with a wild-type R axon projection pattern. Together, this indicates that *bantam* is acting in glial cells to autonomously affect glial cell number and distribution, and cannot reactivate post-mitotic cells.

### *bantam* regulates proliferation of glial cells through *omb*

**Figure 5:**
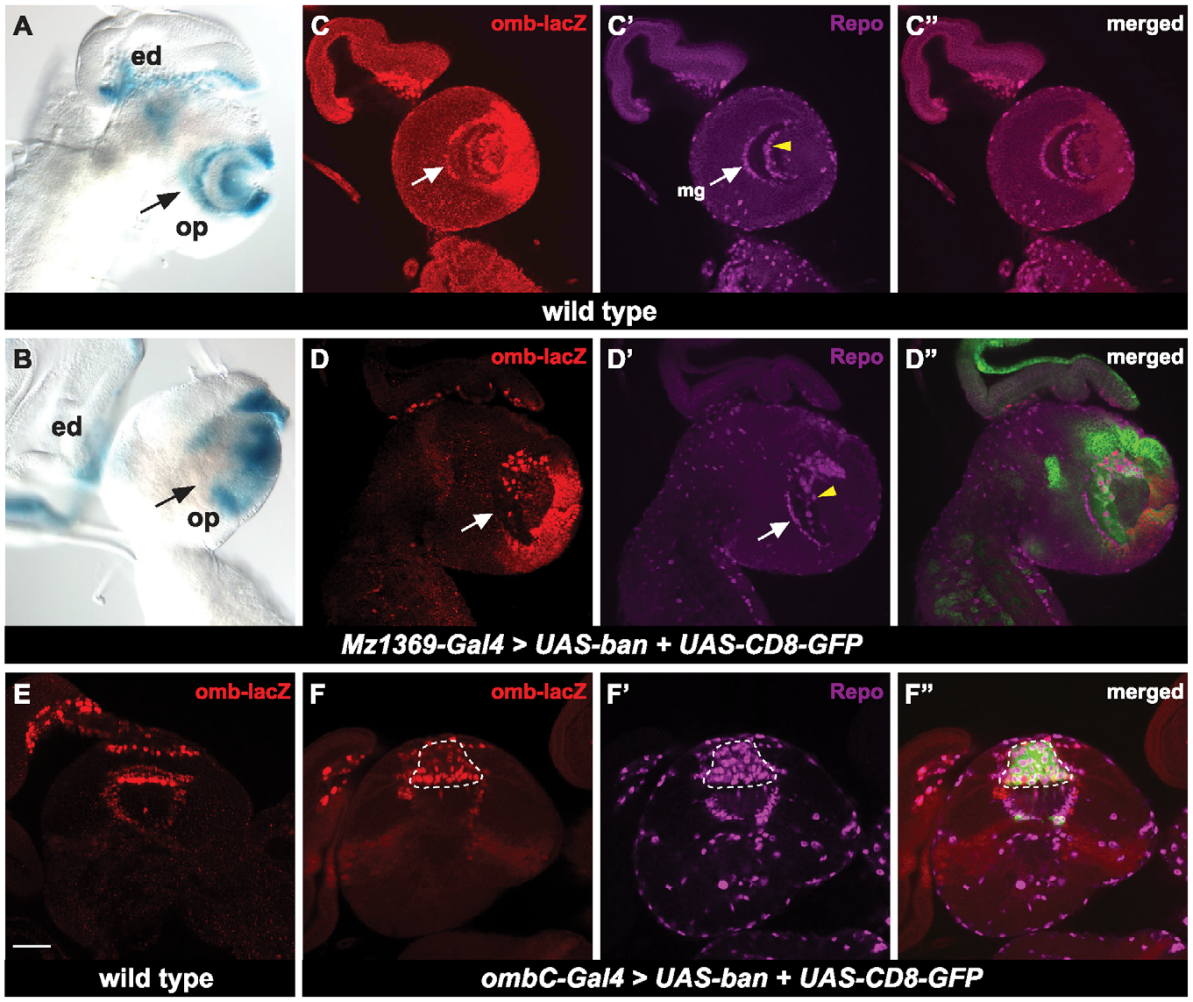
*bantam* down regulates *omb* in the optic lobe. (**A, B**) The brains are positioned for lateral views. X-gal staining indicates *omb-lacZ* expression patterns in the optic lobe. eye disc (ed), optic lobe (op). (**A**) wild type brains; (**B**) over expression of *bantam* by the optic lobe driver, Mz1369-Gal4. (**C–D″**) A single focal plane of lateral view. *UAS*-CD8-GFP (green) is used to view expression of Gal4. Anti-β-galactosidase (red) is to view expression of *omb-lacZ*. Glia cells are viewed by anti-Repo (megenta). (**C–C″**) wild type; (**D–D″**) over expression of *bantam* and *UAS*-CD8-GFP by the optic lobe driver, Mz1369-Gal4. (**C–C″**) and (**D–D″**) are at a similar focal plane. Medulla glia cells are indicated by white arrows and medulla neuropile glial cells are indicated by yellow arrow heads. (**E–F″**) A single focal plane of a horizontal view is shown. (**E**) wild type brains; (**F–F″**) over expression of *bantam* and *UAS*-CD8-GFP by *ombC*-Gal4. Increased glial cells are noted by a dashed line. Scale bar: 50 µm.

*omb* is known to be expressed in glial cells and is important for axonal projections [32] and since *bantam* and *omb* are expressed in glial cells, we wondered if *bantam* regulates *omb* in this developmental context. We used the enhancer trap line, *omb-lacZ*, as a marker for *omb* expression in the optic lobe. *omb-lacZ* [33] is inserted 1.4 kb upstream of the 5′ end of full-length *omb* cDNA [34]. In wild-type animals, *omb-lacZ* showed consistent expression patterns in the optic lobe when compared to *in situs* [35], with high expression in the GPC regions, some differentiated glial cells in the lamina, and in the medulla (Figure 5A, 5C). When *bantam* was over expressed by Mz1369-Gal4, *omb* expression was greatly decreased or totally abolished in most lamina and medulla glial cells (Figure 5B, 5D), showing that *bantam* regulates *omb* expression.

**Figure 6:**
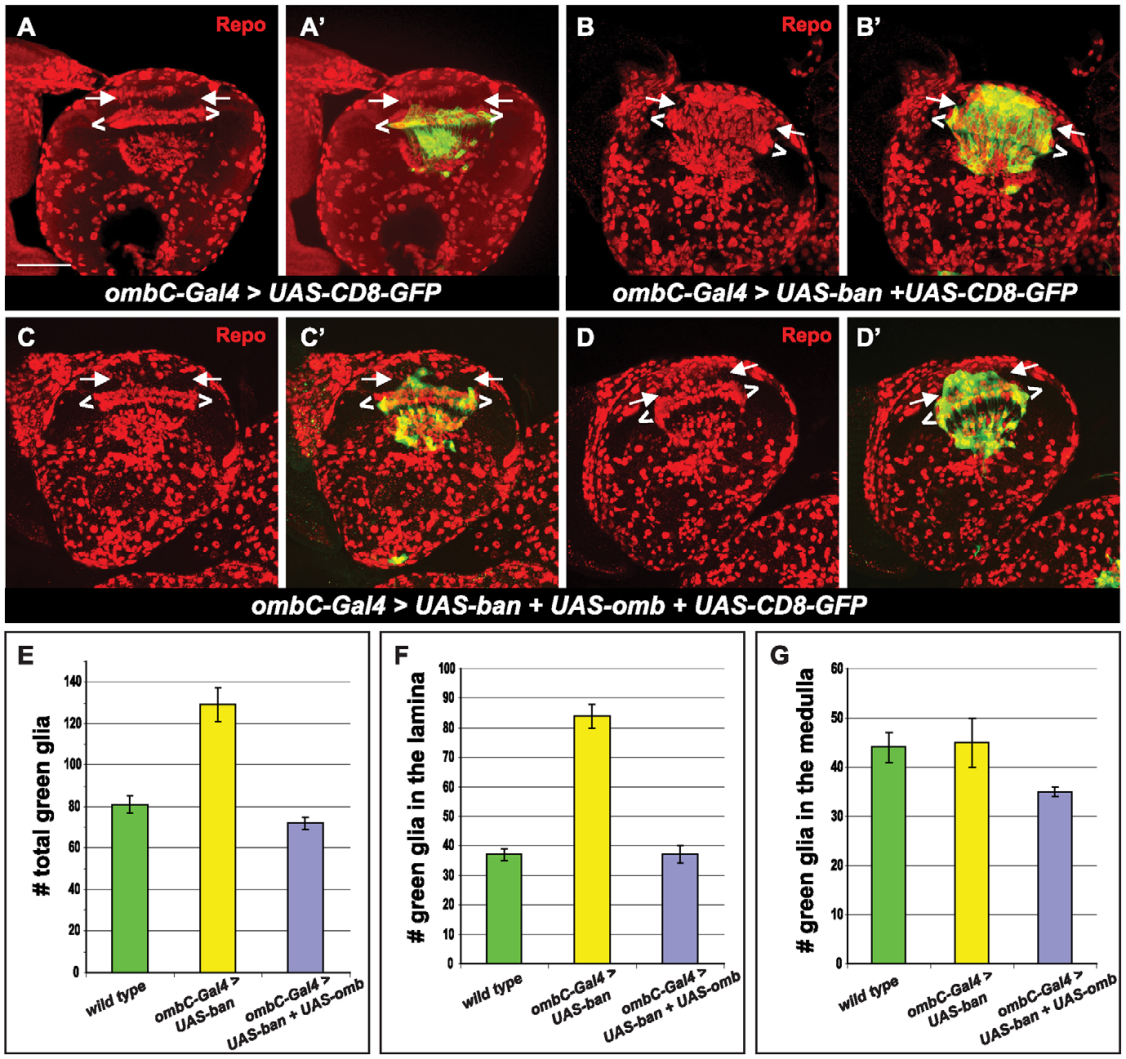
*omb* rescues *bantam*. All brains are positioned for a horizontal view. All pictures are maximum projections from multiple sections. Anti-Repo staining (red) is used to label glial cells. *ombC*-Gal4 expression is visualized by *UAS*-CD8-GFP (green). Glial cells located between white brackets (< >) correspond to the three layers of laminal glial cells: epithelial, marginal and medulla glia. Glial cells in the lamina are located between two arrows. Genotypes: (**A, A′**) *UAS*-CD8-GFP/+; *ombC*-Gal4/+; (**B, B′**) *UAS*-CD8-GFP/+; *ombC*-Gal4/*UAS-ban*; (**C–D′**) *UAS-omb/UAS*-CD8-GFP; *ombC*-Gal4/*UAS-ban*. (E–G) Histograms of green glia number in the optic lobe of the third instar larvae. (E) total green glia in the optic lobe; (F) the green glia in the lamina; (G) the green glia in the medulla. Scale bar: 50 µm.

The next question we asked was whether the regulation of glial cells by *bantam* is dependent on the down-regulation of *omb*. We asked whether expression of *omb* is capable of rescuing the glial cell phenotype caused by *bantam* over expression. We determined that we could not use Mz1369-Gal4 to express both *UAS-ban* and *UAS-omb* as their expression results in embryonic lethality. Instead we used a specific glial cell driver, *ombC*-Gal4 [32], which only expresses in medulla glial cells (meg), located at the base of the lamina plexus at the border of the lamina and the medulla, and in the medulla neuropil glial cells (mng), which enwrap the neuropil in the medulla (Figure 6A and 6A′). When *bantam* was over expressed by *ombC*-Gal4 (Figure 6B, 6B′), the number of glial cells significantly increased (Figure 6E, p-value<0.0005) compared to the wild type. This increase is due to the accumulation of ectopic glial cells in the lamina (Figure 6F), rather than a change of glial cell number in the medulla (Figure 6G). Those ectopic glial cell clusters were in the position where *dachshund*-positive neurons would normally be found (Figure S2). R axons detoured and bypassed the ectopic glial cell clusters, but the final destination of the R1–R6 axons was not affected (Figure S3): they still stopped at the lamina plexus; however, the line formed by their growth cone was not linear. At the place where ectopic glial cells were present, the lamina plexus line became thinner than the rest of the lamina plexus (Figure S3). By observing the *omb-lacZ* levels in *ombC*-Gal4>*UAS-ban*, we found very low levels of *omb* in ectopic glial cells in the lamina (Figure 5F). To rescue this phenotype, we used *ombC*-Gal4 to over express both *UAS-ban* and *UAS-omb*, and found that both glia cell number and distribution were rescued. The total number of this subtype of glia did not show significant difference from the wild type (Figure 6E). In terms of glial cell distribution, about 66% of brains (n = 15) had almost wild-type like glial cell distribution (Figure 6C), and about 34% of brains (n = 15) had a partially rescuing effect (Figure 6D), showing that less ectopic glial cells were present in the lamina compared to when *bantam* was expressed alone (Figure 6B). These results indicate that regulation of *omb* by *bantam* is important in maintaining glial cell number and distribution in the optic lobe.

## Discussion

Our results provide evidence that *bantam* is important for stem cell maintenance in the optic lobe. First, *bantam* shows high expression in the OPC and GPC areas in the optic lobe, where stem cells are located. Second, *bantam* is critical for cell proliferation in the OPC and GPC areas. *ban^Δ1^/ban^Δ1^* null mutants have smaller brains with a dramatic decrease in the proliferation in the OPC and GPC. On the other hand, *bantam* over expression causes brain size to increase, along with increased proliferation in the OPC and GPC. During development, it is very important to maintain a constant stem cell population while differentiated cells are produced. In Drosophila, the central nervous system is derived from neural stem cells called neuroblasts. The optic lobe neuroepithelia are important as they maintain the pool of optic lobe neuroblasts with symmetric division [22]. Misregulation of the self-renewing capacity of the neuroblasts is related to brain tumors; however, the mechanism underlying the precise regulation of proliferation and differentiation of the neuroepithelia and neuroblasts is not well known. miRNAs are known to be crucial for stem cell maintenance in other tissues. When the miRNA processing machinery is affected by loss of *Dicer-1 (Dcr-1)*, which is essential for generating mature miRNAs from their corresponding precursors [36], stem cells cannot be maintained and are lost rapidly in the Drosophila ovary. These *dcr-1* mutant stem cells are delayed in G1 to S transition [37], [38]. *bantam* was reported to be important for germline stem cell (GSC) maintenance in adult Drosophila [12], [13], but the detailed underlying mechanism remains to be determined. It will be interesting to learn how *bantam* affects the cell cycle machinery of stem cells in the OPC and GPC regions. *bantam* has been known to promote cell proliferation in other tissues as well [4], [9]. The ability of *bantam* to promote cell proliferation in various tissues suggests that *bantam* might target molecules that directly, but negatively, affect cell-cycle machinery. Recently, a report showed that *bantam* targets Mei-P26, which has ubiquitin ligase activity, causing the oncogene c-Myc to degrade in the wing imaginal disc [39]. c-Myc can respond to different growth factors to promote cell proliferation through positive regulation of the transcription factor E2F, which is a common G1-S master regulator, and is involved in regulating the expression of a number of genes required for G1-S progress [40]. Future experiments studying whether *bantam* employs this same mechanism in regulating the cell cycle of stem cells in the optic lobe will be informative.

In our work, we also found that *bantam* is required for glial cell growth in the optic lobe. Glial cell numbers in the optic lobe were greatly increased, in a cell-autonomous manner, by an over expression of *bantam*. Conversely, a loss of *bantam* led to a dramatic decrease in glial cells in the optic lobe. During normal development, development of glial cells in the optic lobe is controlled by both extrinsic and intrinsic mechanisms [20]. Glial cell numbers increase rapidly during the third instar larval stage due to the mitosis of differentiated glia, and, more significantly, the proliferation of precursor cells [41], [42]. *bantam* was found to increase proliferation of both glia precursor cells (Figure 2F) and differentiated glia (Figure 6B). In our work, we also provided evidence that *bantam*'s function on glial cell numbers is dependent on its negative regulation of *omb* in a small subgroup of differentiated glial cells, as evidenced by the ability of *omb* to rescue *bantam*'s effect on glial cell numbers and distribution (Figure 6C, 6D). Omb is a T-box transcription factor, highly conserved in all metazoans [43]. The T-box family appears to play critical roles in development, including specification of the mesoderm and morphogenesis in the heart and limbs [44], [45]. In the Drosophila optic lobe, *omb* is expressed in a subgroup of glial cells that are required for their proper positioning and morphology [32]. However, the downstream targets of *omb* responsible for these functions are not clear. Future experiments to determine if the same mechanism is employed in the brain need to be performed.

We think *bantam* does not affect glial cell differentiation because the loss of *bantam* in null mutants still maintains Repo-positive differentiated glial cells. Transcriptional regulators, such as Glial cells missing (Gcm) and its closely related homolog Gcm2, have been well-studied for their roles in glial cell differentiation in the embryonic and postembryonic nervous system of Drosophila [46], [47], [48], [49]. Gcm/Gcm2 are considered to be at the top of the hierarchy for initiating the differentiation of all glial cells. Their downstream targets for maintaining terminal glial cell differentiation include *repo*, *pointed and tramtrack* [50], [51]. With antibody staining for Repo, we did not see any obvious defects in larvae caused by *bantam*, further supporting the idea that *bantam* increases glial cell numbers independent of Gcm-Repo.

Besides promoting glial cell numbers, *bantam* also affects the mobility of glial cells, as we observed an increase in glial cells located under the lamina furrow, the migrating path for glial cells. When *bantam* was over expressed, the three-layer organization of glial cells was disturbed. R-cell axon-derived signals were reported to be required for glial cell proliferation and migration in the lamina [30]. However, our results demonstrated that glial cell defects by *bantam* are cell-autonomous, as neuronal over expression of *bantam* did not show any affect on glial cells. So far, *nonstop*, which encodes an ubiquitin-specific protease, was the only gene reported to be required in laminal glial cells for migration [31]. Future experiments to determine *bantam*'s target genes responsible for glial cell migration will be of interest.

## Materials and Methods

### Drosophila strains and genetics

*Drosophila melanogaster* were grown on standard media at 25°C. For brain size comparisons, embryos were collected for 12–24 hrs, grown for 120–140 hrs, and wandering third instar larvae were selected for dissection.

Over expression of transgenes was done using the Gal4/UAS system [52], [53]. The following Gal4 drivers were used: Mz1369-Gal4 [29], *ombC*-Gal4 [32], *omb*-Gal4, *repo*-Gal4, *elav*-Gal4, and *eyeless*-Gal4 (Bloomington Drosophila Stock Center, Bloomington, IN). The following *UAS* reporters were used: *UAS-omb* [32], *UAS*-CD8-GFP was used to label only the membrane [54], *GS-bantam*, which contains an insertion of the Gene Search UAS element upstream near the *bantam* gene, allowing *bantam* to be over expressed by Gal4 [55], and *UAS-ban* (obtained from I. Edery, Rutgers University), which contains about 300 bp of the *bantam* gene [56] in the pUAST vector. Because these last two *bantam* lines yielded similar results, we used either in this work. Other fly strains used include a *bantam* sensor that contains *tub-EGFP* with two copies of the *bantam* target sequence cloned in the 3′UTR [9], and *omb-lacZ* [57] for detecting the expression of *omb*.

#### X-Gal staining

Third instar larvae were rinsed and dissected in chilled 1× Ringers solution by pulling them apart and inverting the heads [58]. Larval heads with discs attached were fixed in formalin (Sigma) for 18 minutes and then rinsed once for ten minutes in assay buffer (5 mM KH2PO4, 5 mM K2HPO4, 2 mM MgCl2, 100 mM KCl, 4 mM K3[Fe(III)(CN)6], 4 mM K4[Fe(II)(CN)6)]). They were then incubated in pre-warmed reaction buffer (1.5 mg/ml X-Gal in assay buffer) for four hours. Finally, the samples were rinsed in assay buffer to stop the reaction.

#### Antibody staining

The third instar larvae were dissected in chilled 1× Ringers solution by tearing them in half and inverting the heads. Larval heads attached to the body wall were fixed in formalin (Sigma) for 18 minutes at room temperature. PBST (0.3% Triton X-100 in 1× PBS) was used for the subsequent washing and antibody incubation. The primary antibodies used for staining were from Cappel (rabbit anti-β-GAL, diluted 1∶8000) or from the Developmental Studies Hybridoma Bank (DSHB), and included: rat anti-DE-Cadherin (DCAD2, diluted 1∶20), mouse anti-Repo (8D12, diluted 1∶20), mouse anti-Chaoptin (24B10, diluted 1∶400), mouse anti-Dachshund (mAbdac2-3, diluted as 1∶20). Secondary antibodies were conjugated to Cy3 (diluted 1∶200, Jackson ImmunoResearch Lab.) and Alexa Fluor 633 (diluted 1∶100, Invitrogen). All primary antibodies were diluted in PBST and incubated with tissue samples at 4°C overnight. Secondary antibodies were typically incubated with tissue samples for 2 hours at room temperature. Whole larvae brains were dissected after the secondary antibody incubation, washed, and mounted in a Vectashield mounting medium (Vector Laboratories).

#### EdU staining

Dissected larvae were incubated with EdU (20 µM) at room temperature for 10 minutes, washed with PBS, and fixed in formalin (Sigma HT5011) for 18 minutes. Larvae were stained with the Click-iT EdU Alexa Fluor Imaging kits from Molecular probes (Invitrogen) [59]. After washing, larvae were incubated with Alexa Fluor-594 azide cocktail for 30 minutes at room temperature. After washing, whole brains were dissected and mounted in Vectashield mounting medium.

All confocal images were taken on a Leica SP2 confocal microscope, viewed with LCS image browser, and processed with Adobe Photoshop and Illustrator.

#### Statistics and quantitative analysis of glial cell in the optic lobe

Third instar larval brains were stained and mounted for confocal microscopy. Glial cells were identified by anti-Repo staining in nuclei and cells were outlined with a CD8-GFP marker. Neuroepithelial cells were stained by anti-DE-Cadherin, which is a marker for the structure of the optic lobe. A complete series of optical sections were taken at 1 µm intervals for a three-dimensional depiction of the larval brain. Glial cells were manually counted for each section of the Z stacks covering the same target region for each genotype with ImageJ software. Statistics were performed using the JMP statistical software package (SAS). Data were analyzed using the t-test.

#### Measurement of the GPC region in the optic lobe

Third instar larval brains were stained for EdU and mounted to visualize the lateral view. For each sample, the width of GPC was measured in three different areas by the Leica software, and the average value was used to represent the size of GPC. Six to 14 samples were measured for each genotype. Statistics were performed using the JMP statistical software package (SAS). Data were analyzed with the t-test.

## Supporting Information

Figure S1 ***bantam*** **causes abnormal distribution of glia cells with increased numbers in the optic lobe.** All brains are positioned for a horizontal view. *bantam* is over expressed in the optic lobe by *omb*-Gal4. Glial cell are viewed by the anti-Repo (magenta). Neuroepithelia are labeled by anti-DE-Cadherin (red). Expression of *omb*-Gal4 is visualized by GFP (green). (**A, B, C, D**) are maximum projections from multiple sections. (**E, F, G, H**) are single focal planes showing greatly increased number of glial cells in the optic stalk (yellow arrow heads). (**I, J, K, L**) are single focal planes showing the disorganized glial cells at the base of lamina, and ectopic glial cells in the lamina (white arrows). (**M, N, O, P**) are single focal planes showing increased glial cells under lamina furrow (white arrows). Scale bar: 50 µm.
(TIF)

Figure S2 **Over expression of** ***bantam*** **causes ectopic glial cells in the lamina.** Single focal plane for a horizontal view. *UAS*-CD8-GFP (green) is used to view expression of *ombC*-Gal4. Anti-DAC (magenta) is used to label lamina neurons. DE-cadherin staining (red) is used to view neuroepithelial cells. (**A, B, C, D**) wild type; (**E, F, G, H**) *bantam* is over expressed by *ombC*-Gal4. Ectopic glial cells are present in the lamina (arrows). Scale bar: 50 µm.
(TIF)

Figure S3 ***bantam*** **causes ectopic glial cell clusters in the lamina.** Brains are positioned for a horizontal view. Anti-Chaoptin staining (magenta) is used to view R-cell projection patterns. *UAS*-CD8-GFP (green) is used to visualize expression pattern of *ombC*-Gal4 driver. (**A, B, C**) show a single focal plane. R1-R6 terminate at the correct position at the base of the lamina even though they detour to bypass the glial cell clusters (arrows) in the lamina. (**D, E, F**) shows the maximum confocal projections from multiple sections. The ectopic glial cell cluster is present in the lamina. The entire R axon projection pattern is similar to the wild-type pattern. Scale bar: 50 µm.
(TIF)
