# Supplementary figures and images for: bantam Is Required for Optic Lobe Development and Glial Cell Proliferation
Source: PLoS One. 2012 Mar 8;7(3):e32910. doi: 10.1371/journal.pone.0032910 (PMC3297604; doi:10.1371/journal.pone.0032910)

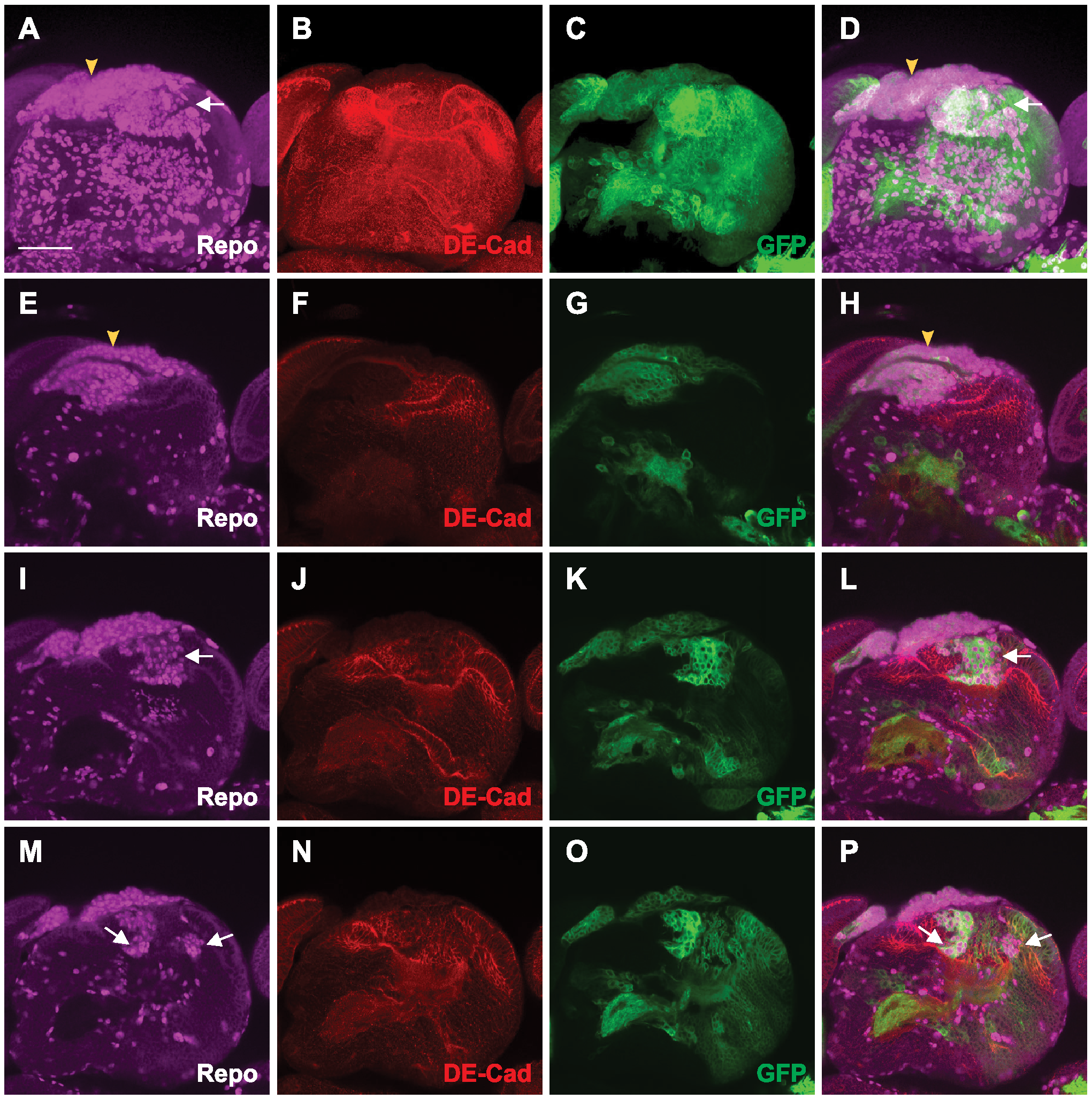

Supplement: Figure S1 — bantam causes abnormal distribution of glia cells with increased numbers in the optic lobe. All brains are positioned for a horizontal view. bantam is over expressed in the optic lobe by omb-Gal4. Glial cell are viewed by the anti-Repo (magenta). Neuroepithelia are labeled by anti-DE-Cadherin (red). Expression of omb-Gal4 is visualized by GFP (green). (A, B, C, D) are maximum projections from multiple sections. (E, F, G, H) are single focal planes showing greatly increased number of glial cells in the optic stalk (yellow arrow heads). (I, J, K, L) are single focal planes showing the disorganized glial cells at the base of lamina, and ectopic glial cells in the lamina (white arrows). (M, N, O, P) are single focal planes showing increased glial cells under lamina furrow (white arrows). Scale bar: 50 µm. (TIF) [file pone.0032910.s001.tif]

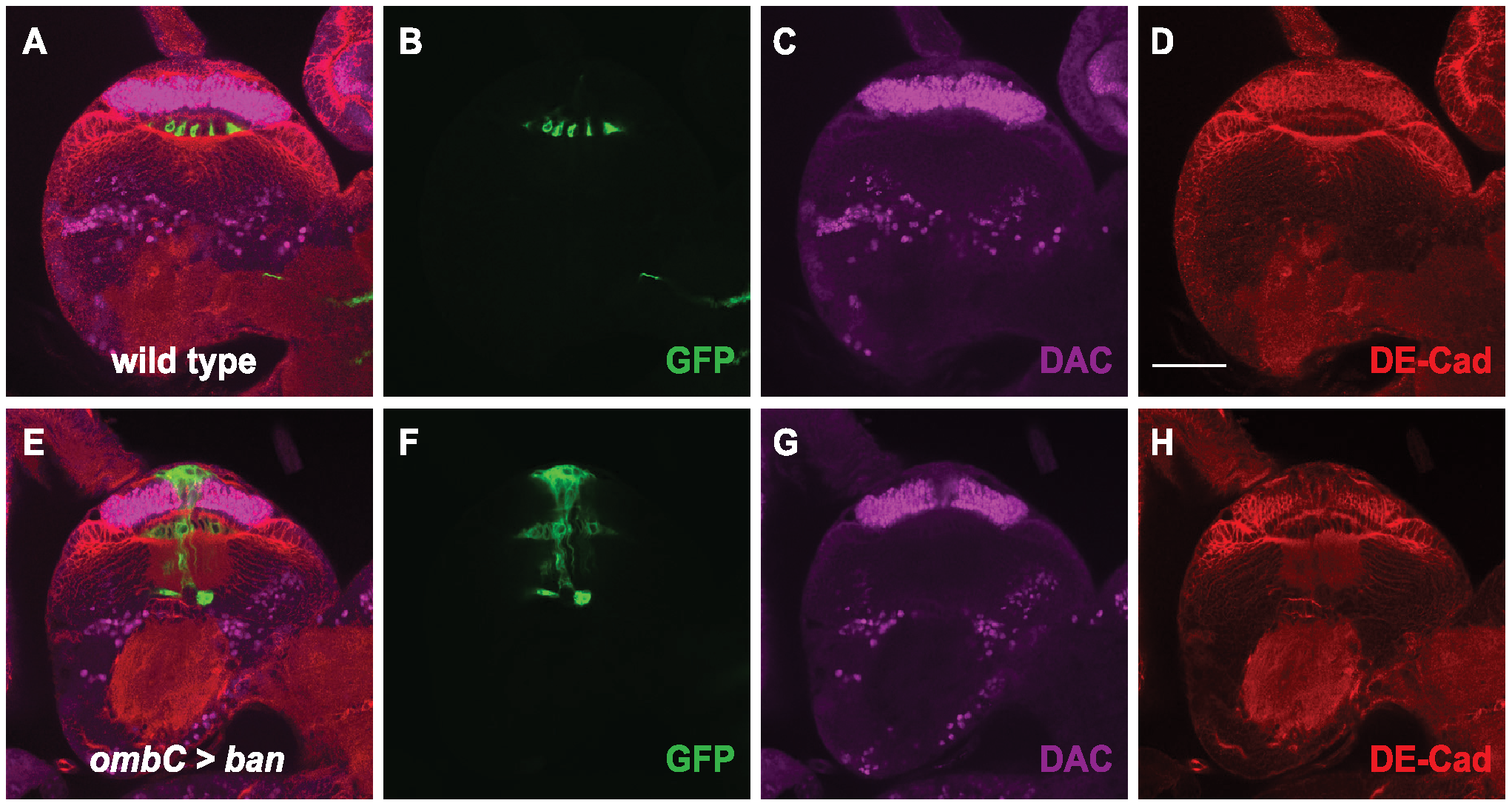

Supplement: Figure S2 — Over expression of bantam causes ectopic glial cells in the lamina. Single focal plane for a horizontal view. UAS-CD8-GFP (green) is used to view expression of ombC-Gal4. Anti-DAC (magenta) is used to label lamina neurons. DE-cadherin staining (red) is used to view neuroepithelial cells. (A, B, C, D) wild type; (E, F, G, H) bantam is over expressed by ombC-Gal4. Ectopic glial cells are present in the lamina (arrows). Scale bar: 50 µm. (TIF) [file pone.0032910.s002.tif]

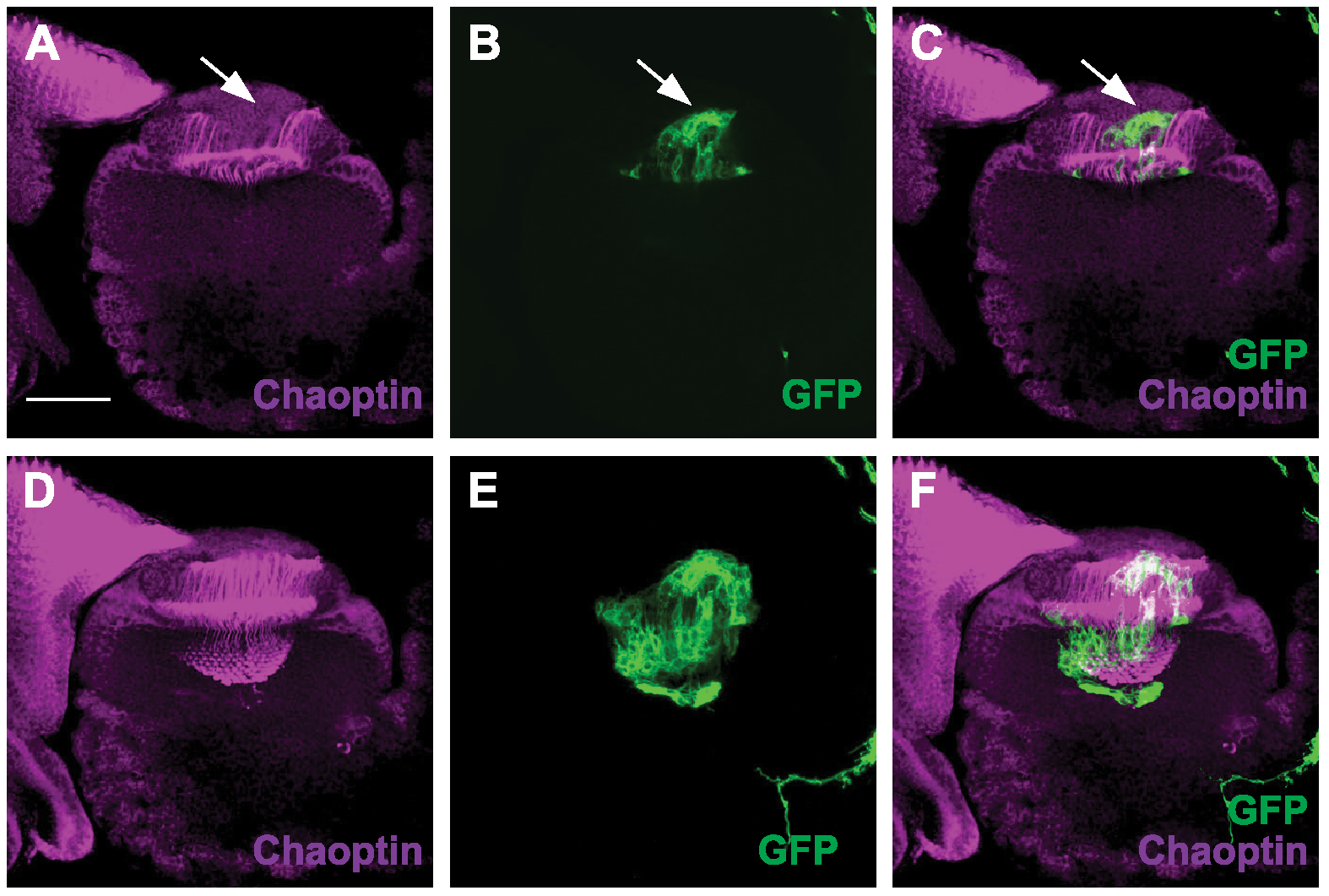

Supplement: Figure S3 — bantam causes ectopic glial cell clusters in the lamina. Brains are positioned for a horizontal view. Anti-Chaoptin staining (magenta) is used to view R-cell projection patterns. UAS-CD8-GFP (green) is used to visualize expression pattern of ombC-Gal4 driver. (A, B, C) show a single focal plane. R1-R6 terminate at the correct position at the base of the lamina even though they detour to bypass the glial cell clusters (arrows) in the lamina. (D, E, F) shows the maximum confocal projections from multiple sections. The ectopic glial cell cluster is present in the lamina. The entire R axon projection pattern is similar to the wild-type pattern. Scale bar: 50 µm. (TIF) [file pone.0032910.s003.tif]
